# Blocking the interaction between interleukin-17A and endoplasmic reticulum stress in macrophage attenuates retinal neovascularization in oxygen-induced retinopathy

**DOI:** 10.1186/s13578-021-00593-6

**Published:** 2021-05-01

**Authors:** Ya’nuo Wang, Shuang Gao, Sha Gao, Na Li, Bing Xie, Xi Shen

**Affiliations:** 1 Department of Ophthalmology, Ruijin Hospital, Shanghai Jiaotong University School of Medicine, 197 Ruijin Er Road, Shanghai, 200025 China; 2 Department of Ophthalmology, Ruijin Hospital, Lu Wan Branch, Shanghai Jiao Tong University School of Medicine, Shanghai, 200025 China

**Keywords:** Endoplasmic reticulum stress, Interleukin-17A, TXNIP/NLRP3 pathway, Retinal neovascularization, Oxygen-induced retinopathy, Ischemic retinopathy

## Abstract

**Background:**

Neovascularization is a leading cause of visual loss typically associated with diabetic retinopathy (DR) and retinopathy of prematurity (ROP). Interleukin-17A (IL-17A) and endoplasmic reticulum (ER) stress both have been demonstrated to play a proangiogenic role in ischemic retinopathies. However, the relationship between IL-17A and ER stress in retinal neovascularization (RNV) under hypoxic conditions and its underlying mechanisms remain unclear.

**Methods:**

In this study, oxygen-induced retinopathy (OIR) mice model was established and intravitreal injections were conducted. Changes of IL-17A and ER stress markers in retinas and cultured primary bone marrow derived macrophage (BMDM) under normoxic or hypoxic conditions were detected. Western blotting, Real-Time RT-PCR, Immunofluorescence assays were conducted to explore the roles and relationship of IL-17A and ER stress in RNV, as well as its underlying mechanisms.

**Results:**

Compared to that in normal controls, IL-17A and ER stress markers were all remarkably increased under hypoxic conditions both in vivo and in vitro. Neutralization or knock out of IL-17A decreased ER stress. ER stress inhibitor 4-phenylbutyrate (4-PBA), attenuated the production of IL-17A, suggesting a positive feedback loop between IL-17A and ER stress. Inhibition of IL-17A or ER stress decreased areas of nonperfusion and neovascularization in OIR retinas. As TXNIP/NLRP3 pathway activation has been demonstrated to be involved in increased retinal vascular permeability of ischemic retinopathy, we observed that TXNIP/NLRP3 pathway mediated in the interaction between IL-17A and ER stress under hypoxic conditions.

**Conclusion:**

The interplay between IL-17A and ER stress contributes to RNV in macrophages via modulation of TXNIP/NLRP3 signaling pathway under hypoxic conditions. The feedback loops may become an innovative and multiple pharmacological therapeutic target for ischemic retinopathy.

**Supplementary Information:**

The online version contains supplementary material available at 10.1186/s13578-021-00593-6.

## Introduction

Retinal neovascularization (RNV) is a primary cause of blindness in several vision-threatening diseases, such as diabetic retinopathy (DR), retinopathy of prematurity (ROP), central retinal vein occlusion (CRVO), age-related macular degeneration (AMD) and neovascular glaucoma (NVG), which occurs when retinal blood supply is insufficient to meet the metabolic demands under the stimulation of ischemia or hypoxic conditions [1–3]. Currently, anti-vascular endothelial growth factor (anti-VEGF) agents and laser photocoagulation are the most effective therapies for RNV [4]. However, the long-term effects of anti-VEGF agents and laser photocoagulation remain unclear, with reported they can cause some side effects [5] and fail to address the potential neurovascular damage [6]. Thus, identifying new and effective pharmacological strategies for targeting RNV is urgently needed.

Recent years, accumulating evidences revealed that Interleukin 17A (IL-17A, commonly known as IL-17) mediates neovascular progression in some diseases [7, 8]. IL-17A is a signature cytokine belongs to IL-17 family, produced by T helper 17 (Th17) cells, a subset of CD4^+^ T cells [9]. The IL-17 family consists of IL-17A, IL-17B, IL-17C, IL-17D, IL-17E and IL-17F [10]. In which, IL-17A is the most widely investigated cytokine for its pro-inflammatory role in several diseases [11, 12]. In addition to Th17 cells, IL-17A is also produced in γδ T cells and macrophages [13]. In fact, besides vascular injury, inflammation is involved in the pathological process of ischemic retinopathies, and inflammation could facilitate the formation of new vessels [14]. In aqueous humor of patients with DR, the levels of inflammatory cytokines IL-1β, IL-6, IL-8, IL-17A, and TNF-α were increased in parallel with the progression of neovascularization [15]. Retinal inflammation following ischemic retinopathy is characterized by activated inflammatory cell accumulation and activation, including Müller cells and macrophages [16]. They can release inflammatory factors, such as IL-17A [17], IL-1β, TNF-α and MCP-1 [7, 16]. Exogenous IL-17A increased ocular NV by promoting M1 (pro-inflammatory) and mitigating M2 (anti-inflammatory) macrophage polarization [7], which further heightened retinal inflammatory responses.

Furthermore, endoplasmic reticulum (ER) stress is a critical factor contributing to inflammation and neovascularization in retinas under ischemia and hypoxia [18–20]. ER, one of the largest organelles in eukaryotic cells, is responsible for protein synthesis and processing, as well as calcium homeostasis and lipid biosynthesis [21]. Dysregulated protein processing due to various physiologic and pathologic conditions, such as nutrient or glucose deprivation, type II diabetes and virus infection, causes protein misfolding and misfolded proteins accumulation in the ER, leading to ER stress [22, 23]. Activating transcription factor 4 (ATF4), glucose-regulated 78 kDa protein (GRP78) and C/EBP homologous protein (CHOP) are key markers of ER stress [23]. Notably, compelling evidence suggested that ER stress was a critical factor contributing to the inflammation and neovascularization in retinas under ischemia and hypoxia [18–20]. Additionally, TXNIP/NLRP3 pathway activation has been demonstrated to be involved in increased retinal vascular permeability of ischemic retinopathy [24], and ER stress could induce NLRP3 inflammasome activation by IRE1α and PERK pathway, regulating release of cytokines such as IL-1β, IL-6, IL-18, TNF-α, MCP-1 and NLRP3, which is the pathological basis of various inflammatory diseases [25, 26]. The role of TXNIP in regulating NLRP3 inflammasome signaling by IRE1α was also demonstrated [27]. However, as to wheather ER stress could activate TXNIP/NLRP3 pathway and successively leads to a series of inflammatory cascades in ischemic retinopathy, data is scarce.

Taken together, given the contribution of IL-17A and ER stress to inflammation and neovascularization processes, is there any association between IL-17A and ER stress in the pathological process of ischemic retinopathy ? Hopefully, the relationship of IL-17A and ER stress has been revealed in several diseases [11, 28–30]. However, data is scarce on retinopathy about their relationship. Therefore, we investigated the relationship between IL-17A and ER stress in OIR model of wild type (WT) or IL-17A knock out (KO) mice for the first time, as well as in macrophages cultured under hypoxia. Furthermore, we explored the potential mechanisms mediated the interplay between IL-17A and ER stress in macrophages under hypoxia in vivo and in vitro.

## Results

### Alterations of IL-17A and ER stress markers in OIR retinas and macrophages under hypoxic conditions

**Fig. 1:**
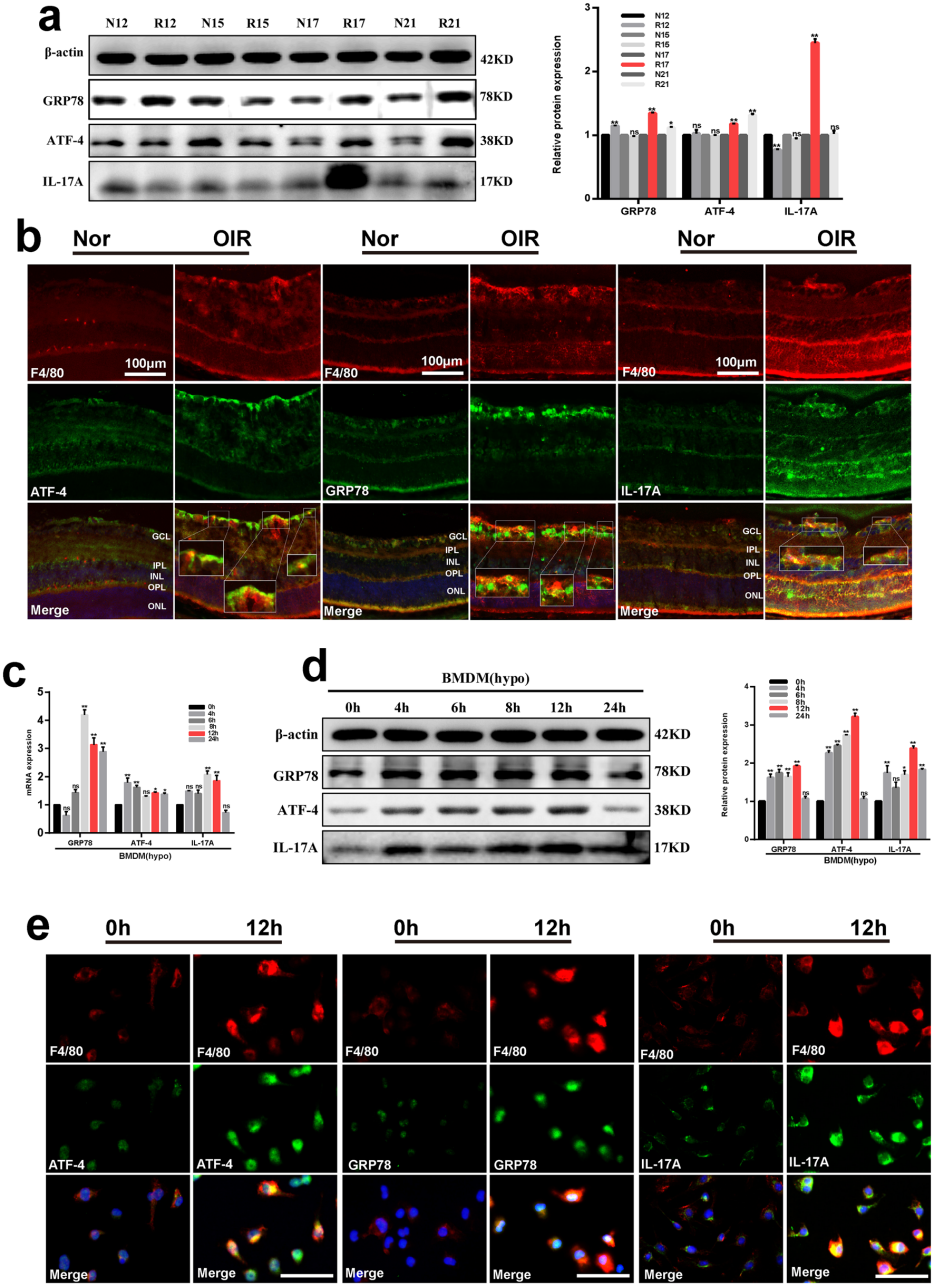
Hypoxia-induced changes of IL-17A and ER stress markers in retinas and macrophages. **a** Western blotting analysis of GRP78, ATF-4 and IL-17A protein levels at P12, P15, P17, P21 in retinas of OIR mice compared with their age-matched controls. **b** Immunofluorescent staining of F4/80 (red, specific expressed by macrophage) and ATF-4 (green), GRP78 (green), IL-17A (green) on representative sections of retinas from P17 OIR mice and normal controls. Boxed areas are magnified in the images. Yellow represents co-localization of GRP78, ATF-4 or IL-17A and macrophages infltration. Scale bars, 100 μm. **c, d** Analysis of mRNA (**c**) and protein (**d**) levels of GRP78, ATF-4 and IL-17A in macrophages exposed to hypoxia for 0, 4, 6, 8, 12 or 24 h. **e** Immunofluorescent staining of F4/80 (red) and ATF-4 (green), GRP78 (green), IL-17A (green) in macrophages cultured under hypoxic conditions for 0 and 24 h. Yellow represents cellular localization of GRP78, ATF-4 or IL-17A. Scale bars, 50 μm. β-actin served as an endogenous reference for normalization. Data are shown as mean ± SEM, n = 6–8 per group for Real-time RT-PCR, n = 3 per group for western blotting. Each experiment repeated three times. ns, no significance. * P < 0.05 and **P < 0.01 compared with control groups. ONL, outer nuclear layer; OPL, outer plexiform layer; INL, inner nuclear layer; IPL, inner plexiform layer; GCL, ganglion cell layer

**Fig. 2:**
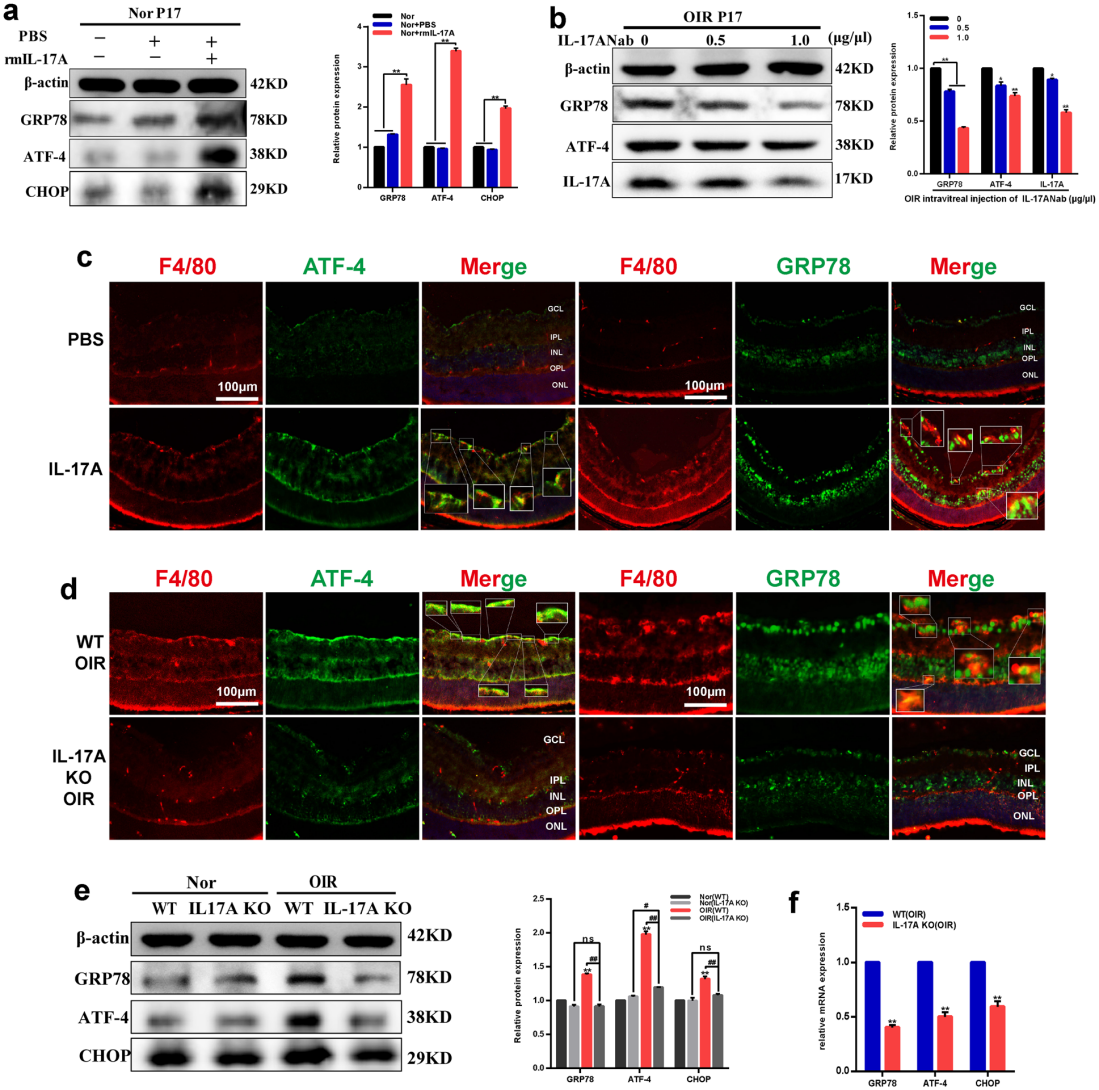
Neutralization or knockout of IL-17A inhibited ER stress in OIR retinas. **a** Effects of rmIL-17A (100 ng/μl) stimulation on the protein levels of GRP78, ATF-4 and CHOP in retinas from WT normal mice. **b** Effects of different concentrations of IL-17ANab (0, 0.5, 1.0 μg/μl) on GRP78, ATF-4 and IL-17A protein levels in retinas from WT-OIR mice. **c** Immunofluorescent staining of F4/80 (red) and ATF-4 (green), GRP78 (green) on representative sections of retinas from normal WT mice with PBS or rmIL-17A stimulation. **d** Immunofluorescent staining of F4/80 (red) and ATF-4 (green), GRP78 (green) in retinas of OIR mice from WT ones and IL-17A KO ones. Boxed areas are magnified in the images. Yellow represents co-localization of GRP78 or ATF-4 and macrophages infltration. Scale bars, 100 μm. **e, f** Changes of retinal GRP78, ATF-4 and CHOP protein (**e**) and mRNA (**f**) levels in OIR mice from WT ones and IL-17A KO ones. β-actin served as an endogenous reference for normalization. Data are shown as mean ± SEM, n = 6–8 per group for Real-time RT-PCR, n = 3 per group for western blotting. Each experiment repeated three times. ns, no significance. * P < 0.05 and **P < 0.01 compared with control groups, ^##^P < 0.01 compared with each other. ONL, outer nuclear layer; OPL, outer plexiform layer; INL, inner nuclear layer; IPL, inner plexiform layer; GCL, ganglion cell layer

IL-17A and ER stress play important roles in promoting angiogenesis of ischemic diseases [15, 18, 19]. To evaluate their involvement in the macrophages of ischemic retinopathy, we examined the expression levels of IL-17A and ER stress markers in OIR retinas and in macrophages under hypoxia. After retinal tissues and macrophages from different groups were harvested, protein levels of IL-17A and ER stress markers were measured. As shown in Fig. 1a, protein expression levels of IL-17A, GRP78 and ATF-4 were increased significantly and concomitantly at P17 in OIR retinas compared with those in their age-matched controls. And expression levels of IL-17A, GRP78 and ATF-4 mRNA (Additional file 1: Fig. S1a–c) were increased significantly in OIR retinas (P12, P15, P17, P21) compared with those in their age-matched controls. Moreover, immunofluorescence co-localization of F4/80 (specific macrophage marker, red) and GRP78, ATF-4, IL-17A ((Fig. 2b, green) were observed in normal and OIR retinas at P17. Double staining of F4/80 and GRP78, ATF-4, IL-17A showed close association between each other (Fig. 2b, yellow), indicating that cytokines expressed in retinal macrophages. Both ATF-4 and GRP78 positive staining in retinal macrophages (Fig. 1b, magnified boxed areas) were increased in OIR retinas compared with those in normal group at P17. These data suggested that hypoxia-induced increased IL-17A production and ER stress activation in retinal macrophages of OIR mice at P17.

Next, we assessed the expression levels of IL-17A, GRP78 and ATF-4 in macrophages, which were cultured in 1% oxygen to mimic the retina ischemia that occurs in OIR. Results showed that mRNA expression levels of IL-17A was elevated significantly at 8 h and 12 h after exposed to hypoxia, while GRP78 and ATF-4 increased strongly in accordance with IL-17A at 12 h after exposed to hypoxia (Fig. 1c). Western blotting assays indicated that expressions of IL-17A, GRP78 and ATF-4 protein were significantly up-regulated in cells cultured under hypoxic conditions, simultaneously reaching a peak at 12 h (Fig. 2d). Furthermore, immunofluorescence co-localization of F4/80 (Fig. 2e, red) and GRP78, ATF-4, IL-17A ((Fig. 2e, green) were observed in macrophages cultured under normoxic (0 h) and hypoxic (12 h) conditions. Both ATF-4 and GRP78, as well as IL-17A, positive staining in macrophages (Fig. 1e, yellow) were increased after exposed to hypoxia for 12 h compared with those in normoxia group (0 h). These data indicated that hypoxia-induced increased IL-17A production and ER stress activation in macrophages in vitro.

### IL-17A promoted ER stress activation in OIR retinas

To investigate the effects of IL-17A on ER stress, we measured mRNA and protein expression levels of ER stress markers in normal mice retinas with or without rmIL-17A intravitreally injected. The data demonstrated that rmIL-17A treatment increased retinal GRP78, ATF4 and CHOP mRNA (Additional file 1: Fig. S2a) and protein (Fig. 2a) levels compared with that in PBS treatment group (P < 0.01). As to retinas of OIR mice, intravitreal injection of IL-17ANab notably downregulated GRP78, ATF4 and CHOP mRNA (Additional file 1: Fig. S2b–d) and protein (Fig. 2b) levels at the concentration of 1.0 μg/μl compared with that in the PBS treatment group (0 μg/ml) (P < 0.01). Moreover, immunofluorescence co-localization of F4/80 (specific macrophage marker, red) and ATF-4 (Fig. 2c, green), GRP78 (Fig. 2c, green) were observed in WT-normal retinas treatment with PBS or rmIL-17A, as well as retinas of WT-OIR or IL-17A KO-OIR mice. Double staining of F4/80 and ER stress markers showed a close association between each other (Fig. 2c, d, yellow), indicating that ER stress markers expressed in retinal macrophages. Both ATF-4 and GRP78 positive staining in retinal macrophages were increased after intravitreal injection with rmIL-17A compared with that in PBS treatment group (Fig. 2c, magnified boxed areas). However, compared to WT-OIR mice, decreased ATF-4 and GRP78 positive staining in retinal macrophages was observed in IL-17A KO-OIR mice (Fig. 2d, magnified boxed areas). In addition, western blotting analysis showed that compared to WT-OIR mice, retinas of IL-17A KO-OIR mice had significantly decreased expression of GRP78, ATF4 and CHOP both in protein (Fig. 2e) and mRNA (Fig. 2f) levels (P < 0.01). And there was no significant difference was observed in protein levels between normal and OIR group of IL-17A KO mice (Fig. 2e). These data suggested that IL-17A neutralization alleviated hypoxia-induced ER stress activation in retinal macrophages of OIR mice.

### IL-17A promoted ER stress activation in macrophages cultured under hypoxic conditions

**Fig. 3:**
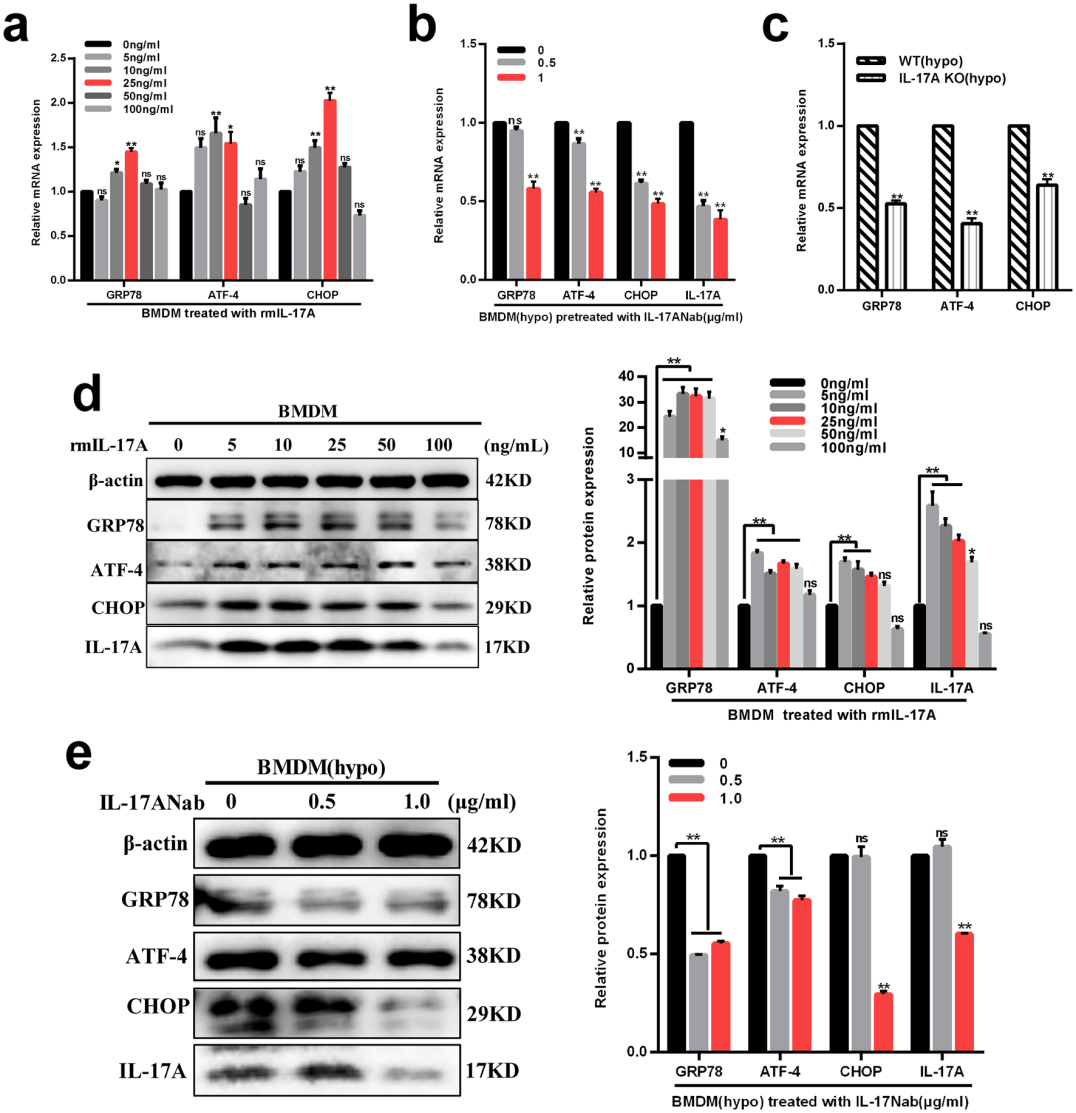
Neutralization or knockout of IL-17A dampened ER stress in macrophages cultured under hypoxic conditions. **a, d** Real-time RT-PCR and western blotting analysis of GRP78, ATF-4 and CHOP mRNA (**a**) and protein (**d**) levels in macrophages pretreated with rmIL-17A for 24 h at different concentrations (0, 5, 10, 25, 50, 100 ng/ml). **b, e** Real-time RT-PCR and western blotting analysis of GRP78, ATF-4, CHOP and IL17A mRNA (**b**) and protein (**e**) levels in macrophages exposed to hypoxia for 12 h pretreated with IL-17ANab at different concentrations (0, 0.5, 1.0 μg/ml). **c** Changes of GRP78, ATF-4 and CHOP mRNA levels in macrophages from WT mice and IL-17A KO mice cultured under hypoxic conditions for 12 h. β-actin served as an endogenous reference for normalization. Data are shown as mean ± SEM, n = 6–8 per group for Real-time RT-PCR, n = 3 per group for western blotting. Each experiment repeated three times. ns, no significance. * P < 0.05 and **P < 0.01 compared with control groups

To detect whether IL-17A induced ER stress in macrophages in vitro, we analyzed the levels of ER stress markers in macrophages treated with different concentrations of rmIL-17A for 24 h. The results demonstrated that there were simultaneous and significant up-regulation of GRP78, ATF4 and CHOP mRNA expression in 10 ng/ml and 25 ng/ml rmIL-17A treatment groups compared with those in PBS treatment group (0 ng/ml) (P < 0.05) (Fig. 3a). Western blotting analysis showed that ER stress markers accompanied with IL-17A protein expression levels were increased significantly in groups of 5 ng/ml continue to 25 ng/ml rmIL-17A treatment compared with those in PBS treatment group (0 ng/ml) (P < 0.01) (Fig. 3d). Therefore, the concentration of 25 ng/ml rmIL-17A was selected as a positive control of providing maximal stimulation of cytokine expression. Additionally, IL-17A neutralization significantly attenuated GRP78, ATF4 and CHOP mRNA (Fig. 3b) and protein (Fig. 3e) levels in macrophages cultured under hypoxia for 12 h at the concentration of 1.0 μg/ml compared with those in PBS treatment group (0 μg/ml) (P < 0.01) (Fig. 3b, e). Moreover, we detected levels of ER stress markers in primary BMDMs from WT and IL-17A KO mice following exposure to hypoxia for 12 h. Real-time RT PCR results showed a significant decrease in the expression levels of GRP78, ATF4 and CHOP in cells from IL-17A KO mice than those from WT ones (P < 0.01) (Fig. 3c). Taken together, these results suggested that IL-17A implicated in promoting ER stress activation in macrophages cultured under hypoxic conditions.

### ER stress contributed to high levels of IL-17A expression in macrophages exposed to hypoxia

**Fig. 4:**
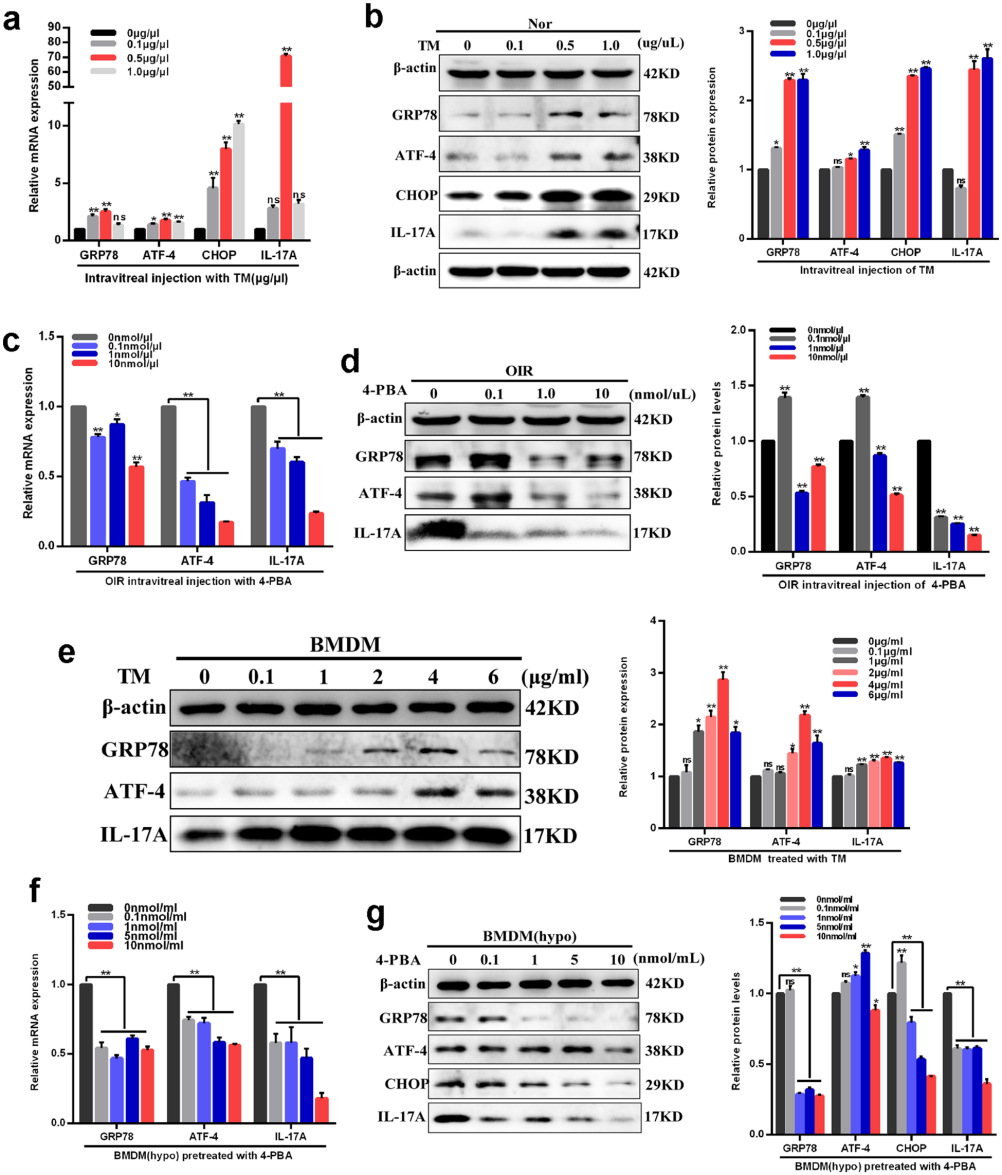
ER stress promoted production of IL-17A in macrophages exposed to hypoxia both in vivo and in vitro. **a, b** Effects of different concentrations of TM (0, 0.1, 0.5, 1.0 μg/μl) on GRP78, ATF-4, CHOP, IL-17A mRNA (**a**) and protein (**b**) expression levels in normal retinas. **c, d** Effects of different concentrations of 4-PBA (0, 0.1, 1, 10 nmol/μl) on GRP78, ATF-4, IL-17A mRNA (**c**) and protein (**d**) expression levels in OIR retinas. **e** Effects of different concentrations of TM (0, 0.1, 1, 2, 4, 6 μg/ml) on GRP78, ATF-4 and IL-17A protein expression levels in macrophages cultured under normoxic conditions. **f, g** Effects of different concentrations of 4-PBA (0, 0.1, 1, 5, 10 nmol/ml) on GRP78, ATF-4, CHOP, IL-17A mRNA (**f**) and protein (**g**) expression levels in macrophages cultured under hypoxic conditions. β-actin served as an endogenous reference for normalization. Data are shown as mean ± SEM, n = 6–8 per group for Real-time RT-PCR, n = 3 per group for western blotting. Each experiment repeated three times. ns, no significance. * P < 0.05 and **P < 0.01 compared with control groups

To further explore the relationship between ER stress response and IL-17A, IL-17A expression levels were detected in normal mice retinas after treatment with different concentrations of TM, as well as in OIR retinas after treatment with different concentrations of 4-PBA. Results demonstrated that TM treatment simultaneously increased retinal ER stress markers and IL-17A mRNA (Fig. 4a) and protein (Fig. 4b) expression levels at the concentration of 0.5 μg/μl compared with those in vehicle treatment group (P < 0.01) (Fig. 4a, b). As to retinas of OIR mice, 4-PBA intravitreal injection significantly downregulated ER stress markers accompanied with decreased IL-17A mRNA (Fig. 4c) and protein (Fig. 4d) levels at the concentration of 1.0 and 10 nmol/μl compared with those in vehicle treatment group (0 µg/ml) (P < 0.01) (Fig. 4c, d). Therefore, concentrations of 0.5 µg/μl TM and 10 nmol/μl 4-PBA were chosen as the effective treatment concentrations. These findings indicated that in OIR retinas, ER stress activation induced higher level of IL-17A production in retinal macrophages.

To confirm the above results in macrophage in vitro, we analyzed IL-17A levels in macrophages treated with different concentrations of TM for 6 h. It was observed that there was significant upregulated expression of IL-17A, as well as GRP78 and ATF-4, mRNA (Additional file 1: Fig. S3) and protein (Fig. 4e) levels in 2 μg/ml continue to 6 µg/ml (peaked at 4 µg/ml) TM treatment groups compared with those in vehicle treatment group (0 ng/ml) (P < 0.01) (Fig. 4e). Furthermore, 4-PBA significantly alleviated IL-17A, GRP78 and ATF-4 mRNA (Fig. 4f) and protein (Fig. 4g) levels in macrophages cultured under hypoxia for 12 h at a concentration of 10 nmol/ml compared with those of vehicle (0 nmol/ml) (Fig. 4f, g). Thus, 4 μg/ml TM and 10 nmol/ml 4-PBA were selected as the effective treatment concentrations of providing maximal stimulation of cytokine expression. Taken together, these results suggested that ER stress contributes to increased levels of IL-17A expression in macrophages exposed to hypoxia both in vitro and in vivo.

### IL-17A interacted with ER stress contributes to RNV

**Fig. 5:**
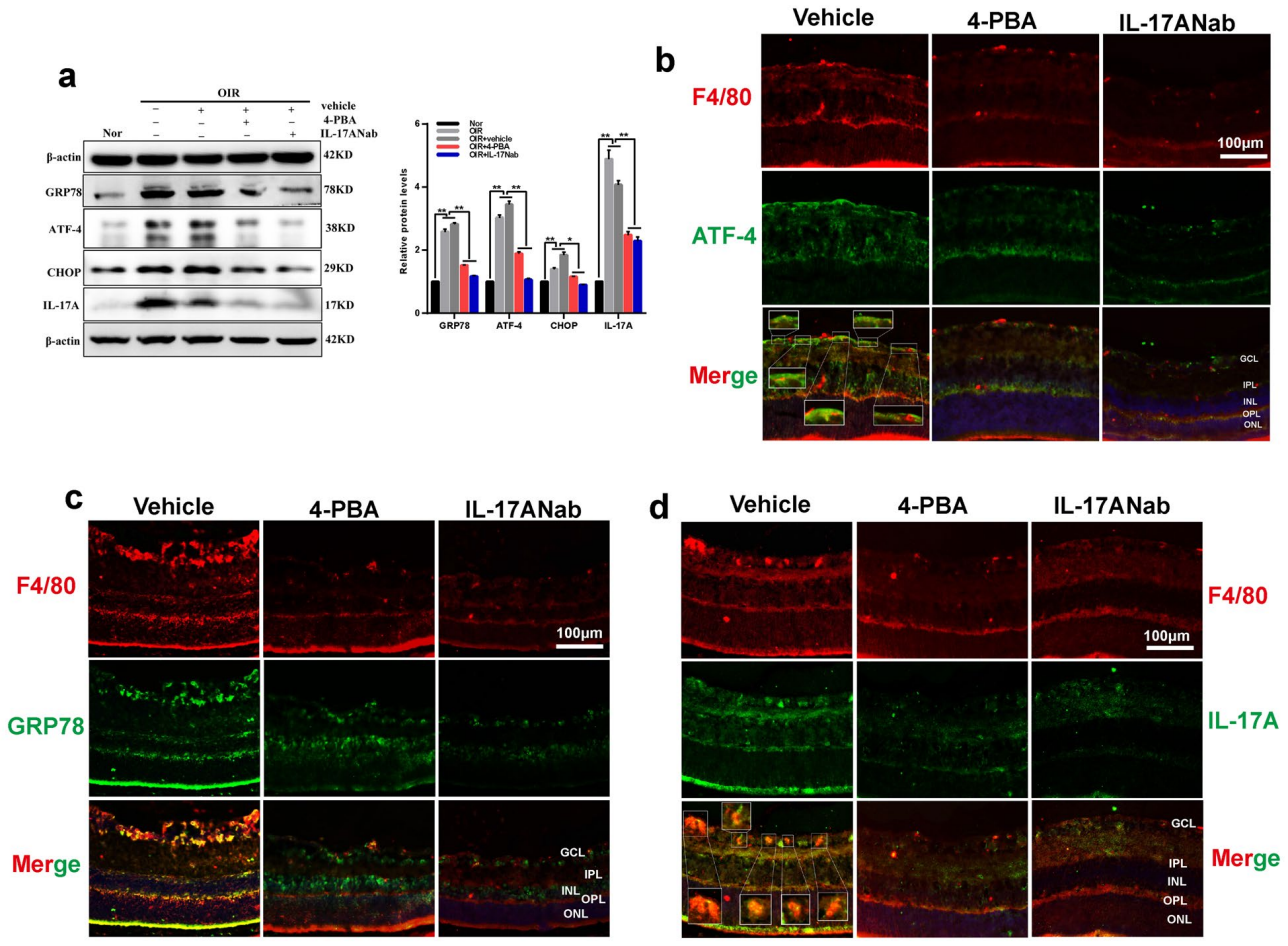
Effects of IL-17ANab or 4-PBA on ER stress markers and IL-17A production in OIR retinas. **a** Western blotting analysis of GRP78, ATF-4, CHOP and IL-17A protein levels in normal retinas and OIR retinas pretreated with 4-PBA (10 nmol/μl) or IL-17ANab (1.0 μg/μl) or vehicle. **b–d** Immunofluorescent staining of F4/80 (red) and ATF-4 (**b**, green), GRP78 (**c**, green), IL-17A (d, green) in OIR retinas treated with 4-PBA or IL-17NAab or vehicle. Boxed areas are magnified in the images. Yellow represents co-localization of ATF-4 or GRP78 and macrophages infltration. Scale bars, 100 μm. β-actin served as an endogenous reference for normalization. Data are shown as mean ± SEM, n = 6–8 per group for Real-time RT-PCR, n = 3 per group for western blotting. Each experiment repeated three times. * P < 0.05 and **P < 0.01 compared with control groups or each other. ONL, outer nuclear layer; OPL, outer plexiform layer; INL, inner nuclear layer; IPL, inner plexiform layer; GCL, ganglion cell layer

To further confirm the interaction between IL-17A and ER stress, we analyzed the expression levels of IL-17A and ER stress markers in OIR retinas, which were pre-treated with 4-PBA or IL-17ANab at their effective concentrations, by western blotting analysis (Fig. 5a) and double immunofluorescence (Fig. 5b–d). Results showed that treatment with 4-PBA or IL-17ANab strongly decreased IL-17A and ER stress markers protein expression levels in OIR retinas, compared with those in vehicle treatment groups (Fig. 5a). Besides, immunofluorescence co-localization of F4/80 (red) and ATF-4 (Fig. 5b, green), GRP78 (Fig. 5c, green), IL-17A (Fig. 5d, green) were observed in OIR retinas intravitreally injected with 4-PBA or IL-17ANab. Double staining of F4/80 and ER stress markers, as well as IL-17A, showed a close association between each other (Fig. 5b–d, yellow), indicating that cytokines expressed in retinal macrophages. Positive staining of ATF-4, GRP78 and IL-17A in retinal macrophages were strongly decreased after treatment with 4-PBA or IL-17ANab compared with those in vehicle treatment group (Fig. 5b–d, magnified boxed areas).

**Fig. 6:**
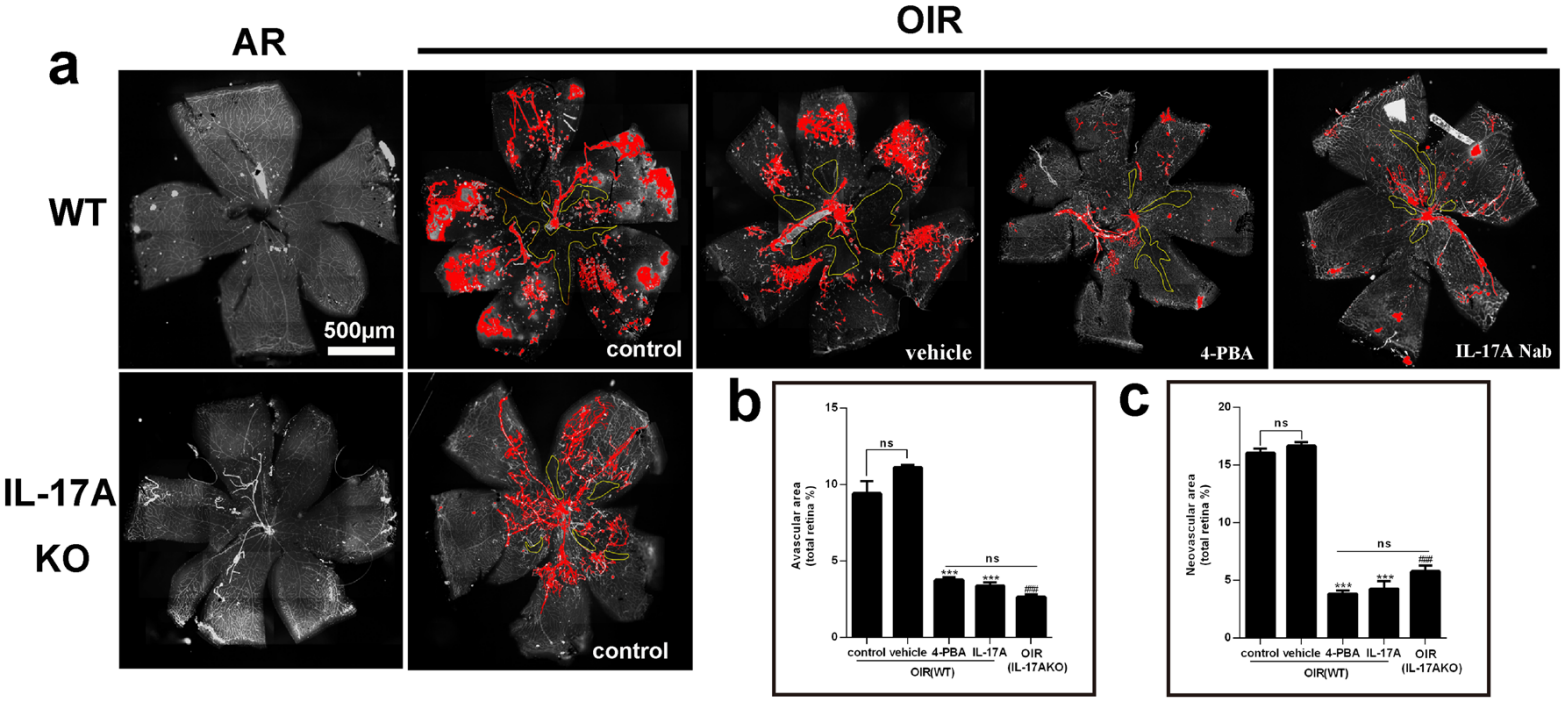
Inhibition of ER stress or IL-17A alleviated RNV. **a** Immunofluorescence staining of fluorescein-conjugated lectin in retinal flat-mounts of normal and OIR WT mice at P17 treated with 4-PBA or IL-17ANab or vehicle, as well as in retinal flat-mounts of normal and OIR IL-17A KO mice. Areas of vaso-obliteration (within yellow lines) and NV (red) were quantified. **b, c** Quantification of the avascular area (**b**) and NV area (**c**) of retinal flat mounts. N = 6 per group. ns, no significance. ***P < 0.001 compared with OIR (WT)-vehicle group. ^###^P < 0.001 compared with OIR (WT)-control group. Scale bars, 500 μm. Data are shown as mean ± SEM. ns, no significance

Next, we evaluated the effects of 4-PBA or IL-17ANab treatment on angiogenesis in OIR mice by analyzing the avascular area and RNV area with isolectin B4 staining in whole-mounted retinas. As a result, 4-PBA or IL-17ANab treatment significantly decreased retinal avascular area by 69.6%, 76.1% and RNV area by 74.4%, 65.3% respectively compared with those in vehicle-treated OIR mice (Fig. 6). It is also worth noting that significantly decreased retinal avascular area by 35.6% and RNV area by 41.9% in retinas from IL-17A KO-OIR mice compared with those in WT-OIR mice (Fig. 6). However, there were no statistical difference among 4-BPA, IL17ANab treatment group and IL-17A KO-OIR group of both avascular area and RNV area.

### Effects of ER stress on activation of TXNIP/NLRP3 pathway

**Fig. 7:**
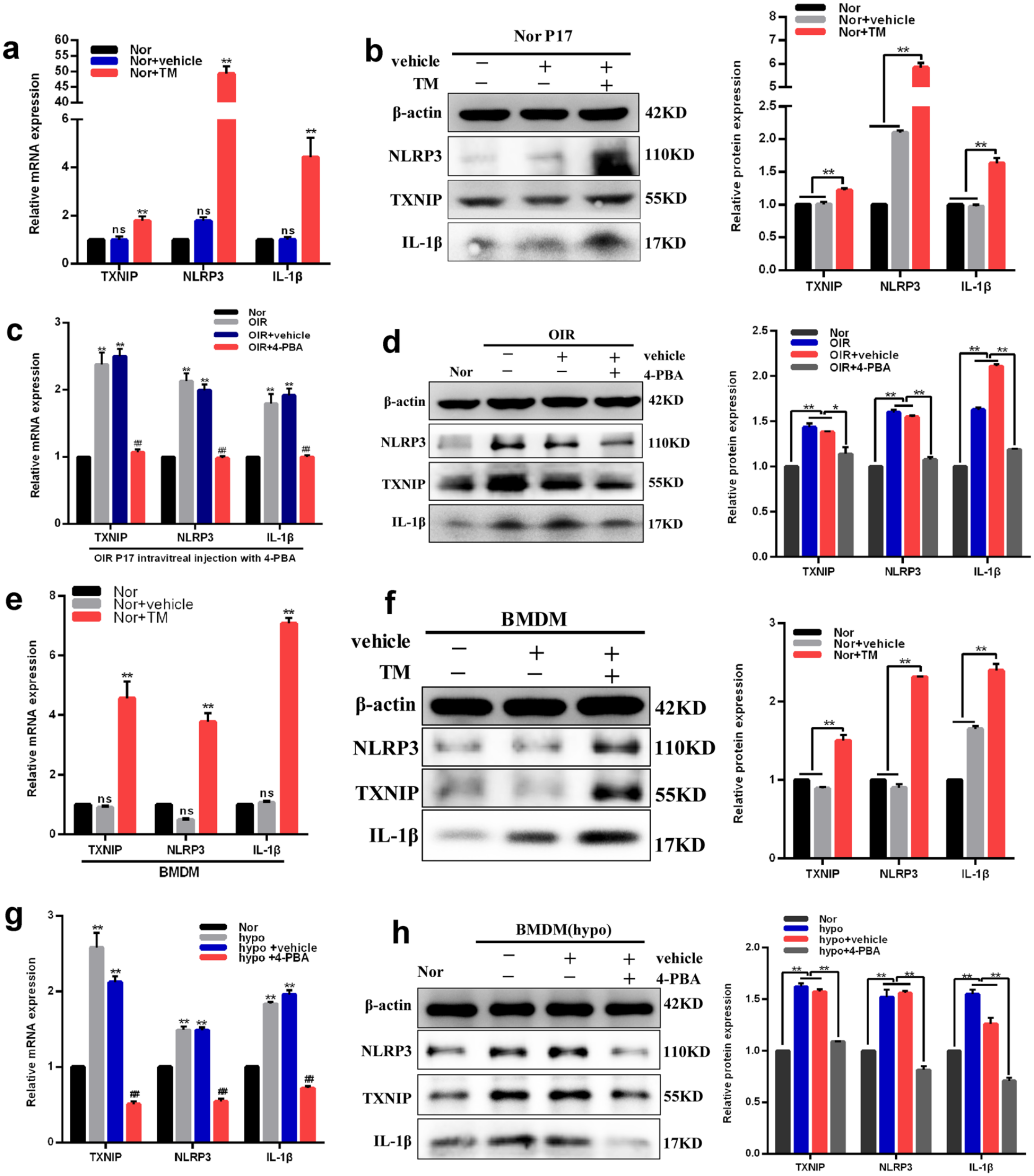
ER stress promoted the activation of TXNIP/NLRP3 pathway in vivo and in vitro. **a, b** Effects of TM (0.5 μg/μl) on TXNIP, NLRP3 and IL-1β mRNA (**a**) and protein (**b**) expression levels in normal retinas. **c, d** Alterations of TXNIP, NLRP3 and IL-1β mRNA (**c**) and protein (**d**) levels in normal and OIR retinas pretreated with 4-PBA (10 nmol/μl) or vehicle. **e, f** Effects of TM (4 μg/ml) on TXNIP, NLRP3 and IL-1β mRNA (**e**) and protein (**f**) expression levels in normal macrophages. **g, h** Changes of TXNIP, NLRP3 and IL-1β mRNA (**g**) and protein (**h**) expression levels in macrophages cultured under normoxic or hypoxic conditions pretreated with 4-PBA (10 nmol/ml) or vehicle. β-actin served as an endogenous reference for normalization. Data are shown as mean ± SEM, n = 6–8 per group for Real-time RT-PCR, n = 3 per group for western blotting. Each experiment repeated three times. ns, no significance. *P < 0.05 and **P < 0.01 compared with control groups or each other

It has previously been known that TXNIP/NLRP3 inflammasome is activated by ER stress in several diseases, sunch as diabetes mellitus, some neurodegenerative diseases and atherosclerosis [31, 32], however, related data is scarce in ischemic retinopathy. To fill the gap, we examined the inflammatory cytokines using western blotting and real-time RT PCR analysis in retinas and macrophages. Results showed that TXNIP, NLRP3 and IL-1β expression levels were strongly upregulated in retinas of mice intravitreally injected with TM (0.5 µg/μl) compared with those in vehicle treatment group (P < 0.01) (Fig. 7a, b). As expected, TXNIP, NLRP3, IL-1β were also overexpressed in OIR retinas compared with those in the normoxia group (Fig. 7c, d). However, intravitreal injection of 4-PBA (10 nmol/μl) significantly decreased the expression levels of TXNIP, NLRP3, IL-1β in the OIR treated group compared with those in the OIR control group (Fig. 7c, d). Furthermore, expression levels of TXNIP, NLRP3, IL-1β in macrophages with different treatments showed similar results with those in retinas (Fig. 7e–h), which indicated that ER stress could stimulate TXNIP/NLRP3 inflammasome activation in the macrophages cultured under hypoxia, as well as in the retinas of OIR.

### Effects of TXNIP/NLRP3 pathway blockage on IL-17A production in retinas and macrophages under hypoxic conditions

**Fig. 8:**
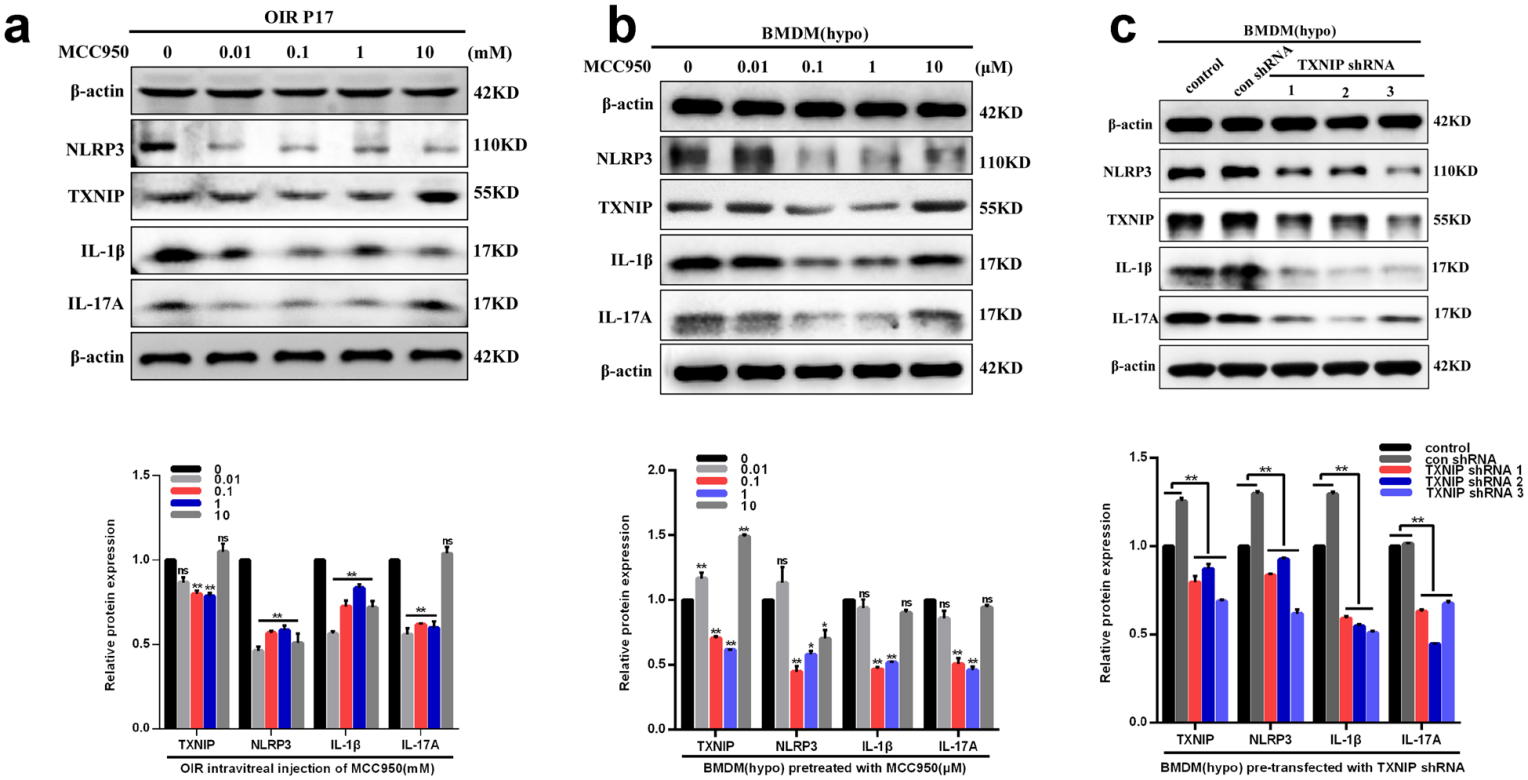
Effects of MCC950 on the production of IL-17A under hypoxia conditions both in vivo and in vitro. **a** Representative immunoblots and densitometric analyses of TXNIP, NLRP3, IL-1β and IL-17A protein expression levels in OIR retinas treated with different concentrations of MCC950 (0, 0.01, 0.1, 1, 10 mM). **b** Representative immunoblots and densitometric analyses of TXNIP, NLRP3, IL-1β and IL-17A protein expression levels in macrophages cultured under hypoxic conditions treated with different concentrations of MCC950 (0, 0.01, 0.1, 1, 10 μM). **c** Representative immunoblots and densitometric analyses showed alterations of TXNIP, NLRP3, IL-1β and IL-17A protein expression levels in macrophages cultured under hypoxic conditions following TXNIP gene silenced with TXNIP shRNA. β-actin served as an endogenous reference for normalization. Data are shown as mean ± SEM, n = 6–8 per group for Real-time RT-PCR, n = 3 per group for western blotting. Each experiment repeated three times. ns, no significance. * P < 0.05 and **P < 0.01 compared with control groups or each other

To assess the effects of TXNIP/NLRP3 pathway blockage by MCC950 on IL-17A production in ischemic retinopathy, western blotting were performed in vitro and in vivo. The data demonstrated that expression levels of IL-17A, as well as NLRP3, TXNIP and IL-1β, significantly decreased after MCC950 intravitreally injected at the concentration of 0.1 mM in retinas of OIR mice compared with those in the OIR control group (0 mM) (P < 0.05) (Fig. 8a). Furthermore, IL-17A expression levels, as well as NLRP3, TXNIP and IL-1β, in the macrophage exposed to hypoxia significantly decreased after treatment with MCC950 at the concentration of 0.1, 1 μM compared with those in the control group (0 μM) (P < 0.05) (Fig. 8b). Besides, western blotting analysis showed that the macrophages, which were cultured under hypoxic conditions following pre-transfected with TXNIP shRNA, expressed significantly decreased levels of IL-17A, as well as TXNIP, NLRP3 and IL-1β, compared with those in control negative shRNA pre-transfected group (Fig. 8c). These results suggested that TXNIP/NLRP3 pathway contributed to IL-17A production in the retina and macrophage under hypoxic conditions.

## Discussion

Abnormal changes of retinal vessels and inflammatory cell infiltration are all-important events in the pathogenesis of ischemic retinopathy. Exploring the interaction mechanisms between neovascularization and inflammation in the pathological process will be conducive to excavate more potential therapeutic modality. In the current research, we found that ER stress contributed to OIR by interplaying with IL-17A in macrophages, and TXNIP/NLRP3 pathway mediated in the interaction between IL-17A and ER stress in promoting neovascularization and inflammation. The identification of the facilitation of RNV by the interaction between IL-17A and ER stress provides a new pharmacological therapeutic target for the treatment of ROP and other ischemic retinopathies.

As known to all, macrophages are crucial adjuster of innate and adaptive immune responses, and have been identified as important angiogenic effector cells that capable of modulating neovascularization [33]. Numerous previous research showed that IL-17A, as a pro-inflammatory cytokine, is involved in macrophage polarization which could induce M1 macrophage polarization but mitigate M2 polarization, by which IL-17A increased RNV in ischemic retinopathy [7, 34]. In this study, results showed that IL-17A was notably increased in retinas of OIR mice at P17, as well as in primary BMDMs cultured under hypoxic conditions for 12 h. Besides, immunofluorescent staining showed that IL-17A expressed in retinal macrophages of OIR models. In line with our findings, previous research [35] revealed that retinal microglia expressed IL-17A in OIR and produced this cytokine in response to hypoxia. Simultaneously, similar to IL-17A, upregulated levels of ER stress markers GRP78 and ATF-4 were observed in macrophages under hypoxic conditions both in vivo and in vitro. Previously, there were studies [20, 36] revealed that ER stress was activated in OIR retinas accompanied with increased expression levels of GRP78, peIF2α, ATF4, and CHOP. However, our results indicated ER stress was activated in retinal macrophages in response to hypoxia for the first time. Despite the revealed contribution of IL-17A and ER stress to retinal macrophages in response to hypoxia respectively, the relationship between IL-17A and ER stress following ischemic retinopathy has not been clarified.

IL-17A augmented ER stress has been implicated in some organ damage, including LPS-induced lung injury [11], intracerebral hemorrhage [28], alcoholic hepatitis [29]. However, data is scarce related to the role of IL-17A on inducing ER stress in retinopathy. In our experiments, we noted that rmIL-17A stimulation significantly upregulated retinal GRP78, ATF-4, CHOP expression in normal mice. However, neutralization of IL-17A decreased the expression of GRP78, ATF-4, CHOP in OIR retinas. Similar results were conducted by using IL-17A KO mice. It is worth noting that immunofluorescence staining showed high coincidence between GRP78, ATF-4 and macrophage. Thus, we further confirmed these results in macrophage in vitro and results showed that IL-17A activated ER stress response at the concentration of 10 ng/ml and 25 ng/ml, while IL-17ANab significantly attenuated high levels of GRP78, ATF4 and CHOP in macrophages exposed to hypoxia at the concentration of 1.0 μg/ml. Similar results were conducted by using cells isolated from IL-17A KO mice. A study from Zhao Yang et al. [28] also demonstrated that IL-17A directly induced ER stress of macrophages in vitro, which lead to macrophage inflammation. However, there was a different effective concentration of IL-17A according to our results. The reason might be that we had different intervention duration. Therefore, unprecedentedly, we conclude that IL-17A may activated ER stress in macrophages of ischemic retinopathy.

In addition to the point IL-17A activated ER stress under some pathological conditions, there were also reports indicated ER stress, as a critical proinflammation factor, promoted the secretion of IL-17A [11, 30, 37]. Therefore, we explored the effects of ER stress on IL-17A production in macrophage both in vivo and in vitro. Our results in vivo demonstrated that elevated IL-17A level was induced by ER stress inducer TM at the concentration of 0.5 and 1.0 μg/μl in normal retinas, accompanying with high levels of ERS markers. However, ER stress inhibitor 4-PBA decreased high levels of IL-17A expression, accompanying with decreased levels of ERS markers, in OIR retinas at the concentration of 10 nmol/μl. Previously, ATF3, as a key transcription factor in ER stress, positively regulated IL-17A production. After infected with S.pneumoniae, ATF3 KO mice showed a marked reduction of IL-17A expression in lung compared to that in WT mice [37]. Also, T lymphocytes and peripheral blood mononuclear cells responded to ERS by activating the IRE1/XBP1 signaling pathway, promoting secretions of IFN-γ, IL-17A, and IL-2 in a study about ulcerative colitis [30]. In mice proximal small intestine, IL-17A production in paneth cells induced by alcohol augmented ER stress [29]. In this study, we further confirmed these results in macrophages in vitro. At concentrations ranging from 2 µg/ml to 6 µg/ml, TM elevated expression levels of IL-17A in macrophages, as well as ER stress markers. 4-PBA, at the concentration of 10 nmol/ml, alleviated high levels of IL-17A and ER stress markers in macrophages cultured under hypoxic conditions. Hence, we conclude that ER stress may promote the secretion of IL-17A in macrophages of ischemic retinopathy for the first time.

Furthermore, treatments with 4-PBA or IL-17ANab at their effective concentrations notably decreased IL-17A and ER stress markers levels in OIR retinas. Inhibition of IL-17A or ER stress also significantly inhibited oxygen-induced macrophage recruitment in retinas as shown by F4/80 immunofluorescence staining. Therefore, it’s reasonable to infer that the interplay between IL-17A and ER stress in retinal macrophages promotes the progression of ischemic retinopathy.

Mounting evidence have demonstrated the role of IL-17A and ER stress in promoting inflammation and neovascularization in ischemic retinopathy respectively. For example, Yanji Zhu et al. [7] reported that IL-17A neutralization alleviated retinal and choroidal neovascularization by promoting M2 and mitigating M1 macrophage polarization. And in DR patients, higher level of IL-17A was observed in parallel with the progression of neovascularization [15]. As to ER stress, a previous report showed that 4-PBA ameliorated inflammation in cultured human retinal endothelial cells exposed to hypoxia, as well as in the retinas of diabetic and OIR mice [18]. Previous study of Li Liu et al. [20] revealed that inhibition of IRE1a, XBP-1, ATF6 each increased VEGF degradation and reduced intracellular VEGF levels, eliciting a reduction of angiogenesis in both the CNV and OIR models in vitro. ER stress inducers TM and thapsigargin (TA) accelerated retinal neovascularization, and GRP78 (BiP) small interfering (si) RNA significantly reduced neovascular outgrowth in mice retinas of OIR model [36]. Consistently, in our current study, 4-PBA and IL-17ANab, as well as knock down of IL-17A, notably dampened retinal avascular area and RNV area in OIR mice model. Immunofluorescence also showed inhibition of IL-17A or ER stress remarkably inhibited hypoxia-induced macrophage recruitments in retinas. Moreover, previous investigations [7] revealed that HUVECs cultured with rIL-17A and rIL-17A-treated macrophage supernatant showed an indirect effect of IL-17A on angiogenesis. And using HUVECs cultured with TM and TM-treated macrophage supernatant, our results showed that TM could play both direct and indirect roles on promoting angiogenesis (Additional file 1: Fig. S4). Previous studies revealed that under ER stress, macrophages are activated and could secrete high levels of inflammatory cytokines sunch as TNF-α, IL-1β, IL-6 [28, 38]. These data suggested that IL-17A and ER stress in macrophages may accelerated RNV in ischemic retinopathy interactionally by promoting macrophage inflammation.

Furthermore, we found TXNIP/NLRP3 pathway was mediated in the interplay between IL-17A and ER stress in OIR. Plenty of studies have shown that TXNIP induced NLRP3 inflammasome activation in endothelial cells, macrophages and pancreatic beta cells in response to reactive oxygen species (ROS) and ER stress in several diseases [25, 31, 32]. Moreover, TXNIP/NLRP3 pathway was activated due to increased ROS production in DR, as well as in Müller cells exposed to high glucose condition [24, 39], indicating its important role in the pathogenesis of ischemic retinopathy. Nevertheless, there is a gap in knowledge concerning whether ER stress leads to the activation TXNIP/NLRP3 pathway in ischemic retinopathy. Consistently, we found TM directly elavated TXNIP, NLRP3 and IL-1β levels, while 4-PBA significantly dampened hypoxia-induced increased expression of TXNIP, NLRP3 and IL-1β both in vivo and in vitro. In addition, we demonstrated the facilitation of IL-17A production by TXNIP/NLRP3 pathway activation using MCC950 (NLRP3 inflammasome inhibitor) and TXNIP shRNA pre-transfected into macrophages cultured under hypoxic conditions. MCC950 is a selective small-molecule inhibitor of the NLRP3 inflammasome, which can specifically inhibit both classical and nonclassical NLRP3 inflammasome activation [40]. These results lend further support to previously documented role of NLRP3 inflammasome activation on IL-17A production [41, 42]. Thus, the present study also fills a gap in knowledge about the relationship between TXNIP/NLRP3 pathway and IL-17A in OIR. Taken together, the evidence points to a crucial role for TXNIP/NLRP3 pathway in the interaction between IL-17A and ER stress in macrophages of ischemic retinopathy.

Finally, some limitations of this study need to be considered. First, we could not determine the effects of ER stress on macrophage polarization, as well as the subsequent macrophage inflammatory cytokines. Second, as there are three signaling pathways involved in ER stress, further studies are needed to identify which one or ones interplay with IL-17A in macrophages of ischemic retinopathy. Therefore, future studies should address this theoretical and empirical gap.

In conclusion, the present study demonstrated a novel mechanism underlying the pathogenesis of ischemic retinopathy. Our findings elucidated IL-17A and ER stress in macrophages are involved in a positive feedback loop, which subsequently promoted ischemic retinopathy by facilitating inflammation and RNV. Moreover, TXNIP/NLRP3 pathway mediated in the interplay between IL-17A and ER stress in macrophages under hypoxic conditions. Therefore, the molecular crosstalk between IL-17A, ER stress and TXNIP/NLRP3 inflammasome activation (Additional file 1: Fig. S5) represents a vicious cycle that can be pharmacologically targeted to curtail RNV in ischemic retinopathy.

## Materials and methods

### Materials and reagents

Dimethylsulfoxide (DMSO) (Cat: D2650), 4-PBA (Cat: P21005) was purchased from Sigma (St.Louis, MO, USA). Recombinant Mouse IL-17A (rmIL-17A) (Cat: 7956-ML) and IL-17ANab (Cat: MAB421) were purchased from R&D Systems (Minneapolis,MN,USA). MCC950 (Cat: S7809) was purchased from Selleck Chemicals (Houston, TX, USA), TM (Cat: ab120296) and primary antibodies against F4/80 (Cat: ab16911), ATF-4 (Cat: ab216839), GRP78 (Cat: ab109659), CHOP (Cat: ab10444), IL-1β (Cat: ab234437), β-actin (Cat: ab179467) were purchased from Abcam (Cambridge, MA, USA), primary antibodies against TXNIP (Cat:14715S), NLRP3 (Cat: 15101S), IL-17A (Cat: 13838S) were purchased from Cell Signaling Technology (Ozyme, SaintQuentin-en-Yvelines, France). Dulbecco’s modified Eagle’s medium (DMEM) (Cat: 10,567,014), Minimum Essential Medium (MEM) α (Cat: 32,561,037), fetal bovine serum (FBS) (Cat:10,099,141) and penicillin–streptomycin (Cat:10,378,016) were obtained from Thermo Scientific (Logan, UT, USA).

### Animal

WT C57BL/6 mice (19–25 g) were purchased from Lingchang Laboratory Animal company (Shanghai, China). IL-17A KO mice (19–25 g) in a C57BL/6 background were purchased from Jackson Labs (The Jackson Laboratory, Bar Harbor, ME, USA). All mice used in this study were pathogen-free. A total of 300 mic were used in this study. The sample size for each experiment was determined based on our previous experiences in similar experimental settings and data published by others. No animals were excluded from the experiments. For each experiment, mice were randomly assigned to different groups according to a randomization procedure (http://www.randomizer.org/). The researchers were blinded to the group allocation during the experiments. All procedures and animal care were performed according to the the guidelines of the Animal Care and Use Committee, with the approval of the Scientific Investigation Board of Shanghai Jiao Tong University, School of Medicine, Shanghai, China. All efforts were made to minimize animal suffering.

To establish OIR mice model [43], postnatal 7 day (P7) pups (4–6 g) and their mothers (19–25 g) were exposed to 75% oxygen for 5 days to P12 and then maintained in room air for an additional 5 days. Oxygen was continuously monitored with an oxygen controller (PRO-OX 110; Reming Bioinstruments Co., Redfield, SD, USA). Mice of normal control group were kept in room air during the entire postnatal period. Pups were sacrificed by cervical dislocation at P12, P15, P17 or P21, and their eyes and retinas were then enucleated.

### Intravitreal injection

At P12, one eye of OIR mice received intravitreal injections of 1 μL 4-PBA (0, 0.1, 1.0, 10 nmol/μl) or 1 μL IL-17ANAb (0, 0.5, 1.0 µg/μl), as well as 1 μL MCC950 ( 0, 0.01, 0.1, 1.0, 10 mM), and the other eye intravitreously injected with 1 μL vehicle. Phosphate buffered saline (PBS) or 0.001% DMSO was used as vehicle control. In addition, one eye of P15 normal mice received intravitreal injections of rmIL-17A (100 ng/μL, 1 μL) [7] and the other eye intravitreally injected with PBS (1 μL). Moreover, one eye of P15 normal mice received intravitreal injections of 1 μL TM (0, 0.1, 0.5 μg/μl) and the other eye intravitreally injected with 1 μL 2% DMSO in PBS (vehicle). Intravitreal injection was performed using a dissecting microscope with a Harvard Pump Microinjection System (Harvard Apparatus, Holliston, MA) and pulled glass micropipettes. All mice were killed at P17, and their eyes and retinas were enucleated.

### Cell culture and treatments

Male C57BL/6 mice (19–25 g) were anesthetized with pentobarbital sodium (50 mg/kg) by intraperitoneal injection. After that, BMDMs were isolated from precursors in the tibias and femurs of the mice as previously described [44]. Bone marrow cells were cultured in α-MEM supplemented with 10% FBS, 100 U/ml penicillin, 100 μg/ml streptomycin in a humidified 5% CO_2_, 37 °C incubator. Hypoxia treatment of cells was as previously described [16]. Briefly, 3 × 10^5^ cells were seeded in each well of 12-well plates. When cells were grown to 80% confluence, media was removed in normoxia and cells were put into a hypoxic chamber (Stem cell technologies, USA) kept at low oxygen tension (1% O_2_, 5% CO_2_ and 94% N_2_, 37 °C). Then cells were cultured in hypoxic chamber for various times with or without different concentrations of 4-PBA (0, 0.1, 1.0, 5, 10 nmol/ml) or IL-17ANab (0, 0.5, 1.0 µg/ml) or MCC950 (0, 0.01, 0.1, 1.0, 10 µM) pretreated. In addition, cells under normoxia were treated with different concentrations of rmIL-17A (0, 5, 10, 25, 50, 100 ng/ml) for 24 h (h) and TM (0, 0.1, 1.0, 2, 4, 6 µg/ml) for 6 h. PBS or 0.001% DMSO was used as vehicle control. Finally, RNA or protein was isolated for real time RT-PCR or western blotting.

### Gene silencing with shRNA

BMDMs were plated into 12-well plate at a density of 3 × 10^5^ cells per well. When cells were grown to 80% confluence, negative control short hairpin RNA (shRNA) plasmids and shRNA plasmid targeting TXNIP (purchased from Shanghai JiKai Gene Chemical Technology Co., LTD.) were transiently transfected to BMDMs with Lipofectamine 3000 plasmid transfection reagent (Cat: L3000015, Invitrogen, USA) and incubated for 24 h.

### Real-time RT-PCR analysis

Total RNA was extracted using Trizol reagent (Cat: 15,596–026, InvitrogenTM, Carlsbad, CA) according to the manufacturer’s protocol, and reversely transcribed into first-strand cDNA using PrimeScript RT Master Mix (Cat: RR036A, Perfect Real Time; Takara, Tokyo, Japan) in a PCR thermal cycler. Quantitative RT- PCR was performed on an ABI Prism 7500-HT system (Applied Biosystems, Foster City, CA) using TB GreenPremix ExTaq (Cat: RR420A, TaKaRa) according to the MIQE guidelines [45]. β-actin was used as an internal control gene. Relative gene expressions were normalized to the expression levels of β-actin, and quantification was performed using the 2^−ΔΔCt^ method. Six or eight samples were used for each analysis and repeated three times. The designed primers used in this study were purchased from Shanghai Shengong Biological Engineering company as follows: β-actin: forward, 5′-GCAGATGTGGATCAGCAAGC-3′ and reverse, 5′-GCAGCTCAGTAACAGTCCGC’; GRP78: forward, 5′-TCATCGGACGCACTTGGAA-3′and reverse, 5′-CAACCACCTTGAATGGCAAGA-3′; ATF-4: forward, 5′-CGGCTGGTCGTCAACCTATAA-3′ and reverse, 5′-GTGGCGTTAGAGATCGTCCT-3′; CHOP: forward, 5′-CATACACCACCACACCTGAAAG-3′ and reverse, 5′-CCGTTTCCTAGTTCTTCCTTGC-3′; IL-17A: forward, 5′-CACCGCAATGAAGACCCTGA-3′ and reverse, 5′-TTCCCTCCGCATTGACACAG-3′; TXNIP: forward, 5′-GACGATGTGGACGACTCTCAAGAC-3′and reverse, 5′-GTTGTTGTTAAGGACGCACGGATC-3′; NLRP3: forward, 5′-GAGCTGGACCTCAGTGACAATGC-3′ and reverse, 5′-ACCAATGCGAGATCCTGACAACAC-3′; IL-1β: forward, 5′-TGCCACCTTTT GACAGTGATG-3′ and reverse, 5′-AAGGTCCACGGGAAAGACAC-3’.

### Western blotting analysis

Retinas and cells were lysed in RIPA buffer (Cat: P0013B, Beyotime, Haimen, China) supplemented with protease inhibitor cocktail (Cat: 78,425, Thermo Fisher Scientific, Waltham, MA) and prepared according to the manufacturer’s instructions. Retinas were triturated by ultrasonication and clarified by centrifugation at 12,000*g* for 20 min at 4 °C, supernatant was collected and protein concentration was determined using the BCA Protein Assay Kit (Cat: 23,227, Thermo Fisher Scientific, Waltham, MA). Samples containing equal amounts of protein were loaded to SDS-PAGE gels and transferred to PVDF membranes (0.45 m, Millipore Co., Ltd.) after electrophoresis. The membranes were blocked with 5% non-fat dry milk which dissolved in 0.05% Tween 20/Tris-buffered saline (TBST) for 2 h at room temperature,and sequentially incubated with primary antibodies at 4℃ overnight. The primary antibodies used were as follows: rabbit anti-GRP78 (1:1000), rabbit anti ATF-4 (1:500), rabbit anti-CHOP (1:1000), rabbit anti-IL-17A (1:1000), rabbit anti-TXNIP (1:1000), rabbit anti-NLRP3 (1:1000), rabbit anti-IL-1-β (1:1000) and rabbit anti-β-actin (1:5000). After washing with TBST, the membrane was incubated with HRP-conjugated secondary antibodies for 2 h at room temperature. Immunoreactivity was visualized by enhanced chemiluminescence (Cat: P0018FM, Beyotime, Haimen, China). The results were quantified using Image J software (U.S. National Institutes of Health, Bethesda, MD, USA) with β-actin as an internal control. Each western blotting replicated three times.

### Immunofluorescence

Eyes of P17 mice were enucleated and fixed using 4% paraformaldehyde (PFA) for 24 h at 4 ℃. Following the removal of the cornea and lens, the eye cup was kept in 30% sucrose overnight at 4 ℃. Subsequently, the eye cup was carefully embedded in optimum cutting temperature (OCT) compound (Cat: C0401, Sakura Global Holdings, Tokyo, Japan) and frozen at − 80 ℃ for cryosections. Sectioning was performed using a cryostat (Leica 840; Leica, Tokyo, Japan). After blocking and permeabilizing, retinal cryosections were incubated with fluorescein-labeled Griffonia simplicifolia lectin I (GSL I) isolectin B4 (Cat: FL-1201, Vector Laboratories, Burlingame, CA, USA) or primary antibodies against F4/80 (rat, 1:50) and GRP78 (rabbit, 1:100), ATF-4 (rabbit, 1:100), IL-17A (rabbit, 1:100) overnight at 4℃, followed by 1 h incubation of fluorescence-conjugated secondary antibodies (1:1000) at room temperature. After washed with PBS, the sections were counterstained with DAPI for nuclear staining and mounted with fluorescence mounting medium (Cat: S3023, Dako, Carpinteria, CA). Retinal cryosections were examined and captured with a fluorescence microscope (Carl Zeiss Micro Imaging, LLC, Oberkochen, Germany).

Eyes of P17 mice were fixed with 4% PFA for 2 h and the retina was dissected from the posterior pole. Retina tissues were incubated with fluorescein labeled Griffonia Simplicifolia Lectin I (GSL I) isolectin B4 according to a previously described method [45]. Retinas were washed with PBS, cutting into 4–6 petals and mounted on microscope slides in Dako Fluorescence Mounting Medium. The flat mounts were imaged with a Zeiss Axiocam HR fluorescent microscope (Germany). The avascular area and neovascular were outlined and highlighted on the images using Adobe Photoshop CS 5 software (Adobe, San Jose, CA, USA) [46]. 6–8 retinas from each group were examined and analyzed.

### Data and statistical analyses

All data were presented as mean ± SEM. The statistical analysis was performed using IBM SPSS Statistics 22 (IBM, Armonk, NY, USA) and GraphPad Prism 6 (Graphpad, San Diego, CA, USA). Values were tested to assess whether they followed a normal distribution by the same software. Comparison between two sets of experiments was analyzed by Student’s t-test while one-way ANOVA was used to determine differences among means in multiple sets of experiments followed by Bonferroni posthoctest. All experiments were independently repeated at least three times. In both cases, statistically significant P-values (< 0.05 and < 0.01) indicated by asterisks (* and **, respectively). ‘ns’ indicates no significance.

## Supplementary Information

**Additional file 1.** mRNA expression, endothelial proliferation and tube formation and ERG in different groups, as well as the proposed pathway scheme.

## Data Availability

All data generated or analysed during this study are included in this article and its Additional file 1.

## Abbreviations

ER stress: Endoplasmic reticulum stress
GRP78: Glucose-regulated 78 kDa protein
ATF-4: Activating transcription factor 4
CHOP: C/EBP homologous protein
IL-17A: Interleukin-17A
TXNIP: Thioredoxin interacting protein
NLRP3: NOD-like receptor family, pyrin domain-containing 3
OIR: Oxygen induced retinopathy
DR: Diabetic retinopathy
ROP: Retinopathy of prematurity
RNV: Retinal neovascularization

## References

[1] Gewaily D, Muthuswamy K, Greenberg PB (2015). Intravitreal steroids versus observation for macular edema secondary to central retinal vein occlusion. Cochrane Database Syst Rev..

[2] Li HY, Yuan Y, Fu YH, Wang Y, Gao XY (2020). Hypoxia-inducible factor-1α: a promising therapeutic target for vasculopathy in diabetic retinopathy. Pharmacol Res.

[3] Liu CH, Wang Z, Sun Y, Chen J (2017). Animal models of ocular angiogenesis: from development to pathologies. Faseb j.

[4] Park YG, Roh YJ (2016). New diagnostic and therapeutic approaches for preventing the progression of diabetic retinopathy. J Diabetes Res.

[5] Day S, Acquah K, Mruthyunjaya P, Grossman DS, Lee PP, Sloan FA (2011). Ocular complications after anti-vascular endothelial growth factor therapy in Medicare patients with age-related macular degeneration. Am J Ophthalmol.

[6] Morin J, Luu TM, Superstein R, Ospina LH, Lefebvre F, Simard MN (2016). Neurodevelopmental outcomes following Bevacizumab injections for retinopathy of prematurity. Pediatrics.

[7] Zhu Y, Tan W, Demetriades AM, Cai Y, Gao Y, Sui A (2016). Interleukin-17A neutralization alleviated ocular neovascularization by promoting M2 and mitigating M1 macrophage polarization. Immunology.

[8] Li Y, Zhou Y (2019). Interleukin-17: the role for pathological angiogenesis in ocular neovascular diseases. Tohoku J Exp Med.

[9] Miossec P, Korn T, Kuchroo VK (2009). Interleukin-17 and type 17 helper T cells. N Engl J Med.

[10] Gaffen SL (2011). Recent advances in the IL-17 cytokine family. Curr Opin Immunol.

[11] Kim SR, Kim HJ, Kim DI, Lee KB, Park HJ, Jeong JS (2015). Blockade of interplay between IL-17A and endoplasmic reticulum stress attenuates LPS-Induced lung injury. Theranostics.

[12] McGeachy MJ, Cua DJ, Gaffen SL (2019). The IL-17 Family of Cytokines in health and disease. Immunity.

[13] Korn T, Petermann F (2012). Development and function of interleukin 17-producing γδ T cells. Ann N Y Acad Sci.

[14] Semeraro F, Morescalchi F, Cancarini A, Russo A, Rezzola S, Costagliola C (2019). Diabetic retinopathy, a vascular and inflammatory disease: therapeutic implications. Diabetes Metab.

[15] Feng S, Yu H, Yu Y, Geng Y, Li D, Yang C (2018). Levels of Inflammatory cytokines IL-1β, IL-6, IL-8, IL-17A, and TNF-α in aqueous humour of patients with diabetic retinopathy. J Diabetes Res.

[16] Gao S, Li C, Zhu Y, Wang Y, Sui A, Zhong Y (2017). PEDF mediates pathological neovascularization by regulating macrophage recruitment and polarization in the mouse model of oxygen-induced retinopathy. Sci Rep.

[17] Qiu AW, Bian Z, Mao PA, Liu QH (2016). IL-17A exacerbates diabetic retinopathy by impairing Müller cell function via Act1 signaling. Exp Mol Med.

[18] Li J, Wang JJ, Yu Q, Wang M, Zhang SX (2009). Endoplasmic reticulum stress is implicated in retinal inflammation and diabetic retinopathy. FEBS Lett.

[19] Geng W, Qin F, Ren J, Xiao S, Wang A (2018). Mini-peptide RPL41 attenuated retinal neovascularization by inducing degradation of ATF4 in oxygen-induced retinopathy mice. Exp Cell Res.

[20] Liu L, Qi X, Chen Z, Shaw L, Cai J, Smith LH (2013). Targeting the IRE1α/XBP1 and ATF6 arms of the unfolded protein response enhances VEGF blockade to prevent retinal and choroidal neovascularization. Am J Pathol.

[21] Saito A, Imaizumi K (2018). Unfolded protein response-dependent communication and contact among endoplasmic reticulum, mitochondria, and plasma membrane. Int J Mol Sci.

[22] Oakes SA, Papa FR (2015). The role of endoplasmic reticulum stress in human pathology. Annu Rev Pathol.

[23] Iurlaro R, Munoz-Pinedo C (2016). Cell death induced by endoplasmic reticulum stress. FEBS J.

[24] Chen W, Zhao M, Zhao S, Lu Q, Ni L, Zou C (2017). Activation of the TXNIP/NLRP3 inflammasome pathway contributes to inflammation in diabetic retinopathy: a novel inhibitory effect of minocycline. Inflamm Res.

[25] Bronner DN, Abuaita BH, Chen X, Fitzgerald KA, Nunez G, He Y (2015). Endoplasmic reticulum stress activates the inflammasome via NLRP3- and caspase-2-driven mitochondrial damage. Immunity.

[26] Hotamisligil GS (2010). Endoplasmic reticulum stress and the inflammatory basis of metabolic disease. Cell.

[27] Sun X, Jiao X, Ma Y, Liu Y, Zhang L, He Y (2016). Trimethylamine N-oxide induces inflammation and endothelial dysfunction in human umbilical vein endothelial cells via activating ROS-TXNIP-NLRP3 inflammasome. Biochem Biophys Res Commun.

[28] Yang Z, Liu Q, Shi H, Jiang X, Wang S, Lu Y (2018). Interleukin 17A exacerbates ER-stress-mediated inflammation of macrophages following ICH. Mol Immunol.

[29] Gyongyosi B, Cho Y, Lowe P, Calenda CD, Iracheta-Vellve A, Satishchandran A (2019). Alcohol-induced IL-17A production in Paneth cells amplifies endoplasmic reticulum stress, apoptosis, and inflammasome-IL-18 activation in the proximal small intestine in mice. Mucosal Immunol.

[30] Li N, Wang XM, Jiang LJ, Zhang M, Li N, Wei ZZ (2016). Effects of endoplasmic reticulum stress on the expression of inflammatory cytokines in patients with ulcerative colitis. World J Gastroenterol.

[31] Lerner AG, Upton JP, Praveen PV, Ghosh R, Nakagawa Y, Igbaria A (2012). IRE1α induces thioredoxin-interacting protein to activate the NLRP3 inflammasome and promote programmed cell death under irremediable ER stress. Cell Metab.

[32] Chen X, Guo X, Ge Q, Zhao Y, Mu H, Zhang J (2019). ER stress activates the NLRP3 inflammasome: a novel mechanism of atherosclerosis. Oxid Med Cell Longev.

[33] Gao X, Wang YS, Li XQ, Hou HY, Su JB, Yao LB (2016). Macrophages promote vasculogenesis of retinal neovascularization in an oxygen-induced retinopathy model in mice. Cell Tissue Res.

[34] Yuan C, Yang D, Ma J, Yang J, Xue J, Song F (2020). Modulation of Wnt/β-catenin signaling in IL-17A-mediated macrophage polarization of RAW264.7 cells. Braz J Med Biol Res..

[35] Talia DM, Deliyanti D, Agrotis A, Wilkinson-Berka JL (2016). Inhibition of the nuclear receptor RORγ and Interleukin-17A suppresses neovascular retinopathy: involvement of immunocompetent microglia. Arterioscler Thromb Vasc Biol.

[36] Nakamura S, Takizawa H, Shimazawa M, Hashimoto Y, Sugitani S, Tsuruma K (2013). Mild endoplasmic reticulum stress promotes retinal neovascularization via induction of BiP/GRP78. PLoS ONE.

[37] Lee S, Kim GL, Kim NY, Kim SJ, Ghosh P, Rhee DK (2018). ATF3 Stimulates IL-17A by regulating intracellular Ca(2+)/ROS-dependent IL-1β activation during streptococcus pneumoniae Infection. Front Immunol.

[38] Wang Y, Zhou X, Zhao D, Wang X, Gurley EC, Liu R (2020). Berberine inhibits free fatty acid and LPS-induced inflammation via modulating ER stress response in macrophages and hepatocytes. PLoS ONE.

[39] Du J, Wang Y, Tu Y, Guo Y, Sun X, Xu X (2020). A prodrug of epigallocatechin-3-gallate alleviates high glucose-induced pro-angiogenic factor production by inhibiting the ROS/TXNIP/NLRP3 inflammasome axis in retinal Müller cells. Exp Eye Res.

[40] Coll RC, Robertson AA, Chae JJ, Higgins SC, Muñoz-Planillo R, Inserra MC (2015). A small-molecule inhibitor of the NLRP3 inflammasome for the treatment of inflammatory diseases. Nat Med.

[41] Xu KY, Tong S, Wu CY, Ding XC, Chen JL, Ming Y (2020). Nlrp3 inflammasome inhibitor MCC950 ameliorates Obliterative bronchiolitis by inhibiting Th1/Th17 response and promoting treg response after orthotopic tracheal transplantation in mice. Transplantation.

[42] Maher BM, Mulcahy ME, Murphy AG, Wilk M, O'Keeffe KM, Geoghegan JA (2013). Nlrp-3-driven interleukin 17 production by gammadeltaT cells controls infection outcomes during Staphylococcus aureus surgical site infection. Infect Immun.

[43] Smith LE, Wesolowski E, McLellan A, Kostyk SK, D'Amato R, Sullivan R (1994). Oxygen-induced retinopathy in the mouse. Invest Ophthalmol Vis Sci.

[44] Zhang X, Goncalves R, Mosser DM (2008). The isolation and characterization of murine macrophages. Curr Protoc Immunol..

[45] Huggett JF (2020). The digital MIQE guidelines update: minimum information for publication of quantitative digital PCR experiments for 2020. Clin Chem.

[46] Connor KM, Krah NM, Dennison RJ, Aderman CM, Chen J, Guerin KI (2009). Quantification of oxygen-induced retinopathy in the mouse: a model of vessel loss, vessel regrowth and pathological angiogenesis. Nat Protoc.

